# A Comparison of the Attitudes to Influenza Vaccination Held by Nursing, Midwifery, Pharmacy, and Public Health Students and Their Knowledge of Viral Infections

**DOI:** 10.3390/vaccines8030516

**Published:** 2020-09-09

**Authors:** Sylwia Kałucka, Elżbieta Dziankowska-Zaborszczyk, Izabela Grzegorczyk-Karolak, Agnieszka Głowacka

**Affiliations:** 1 Department of Hygiene and Epidemiology, Medical University of Lodz, 90-647 Lodz, Poland; 2 Department of Epidemiology and Biostatistics, Medical University of Lodz, 90-647 Lodz, Poland; elzbieta.dziankowska-zaborszczyk@umed.lodz.pl; 3 Department of Biology and Pharmaceutical Botany, Medical University of Lodz, 90-151 Lodz, Poland; izabela.grzegorczyk@umed.lodz.pl; 4 Department of Developmental Nursing and Health Promotion, Medical University of Lodz, 90-251 Lodz, Poland; agnieszka.glowacka@umed.lodz.pl

**Keywords:** influenza vaccination, student, nursing, public health, pharmacy, midwifery, vaccine, common cold

## Abstract

Influenza is a viral vaccine-preventable disease. The present study aims to explore the attitude to influenza immunization and the knowledge of influenza among students in Poland. A cross-sectional survey was conducted at the end of 2019 and the beginning of 2020 among students of Nursing, Midwifery, Pharmacy, and Public health in all years of study. Data was obtained from 1137 students (90.7% female, 9.3% male), mean age 21.3 ± 1.62 years. The urban students were more likely to be vaccinated against seasonal influenza than rural students (OR: 1.52; 95% CI [1.10–2.10], *p* = 0.010362). The students of Public health were more likely to be vaccinated against influenza (48.9%, regularly annually 1.1%) than Pharmacy (31%, regularly annually 2.5%), Nursing (30.7%, regularly annually 1.3%) or Midwifery (25.1%, regularly annually 2.4%). First-year and second-year students were vaccinated more often (OR: 2.75; 95% CI [1.99–3.82], *p* = 0.00000; OR: 1.84; 95% CI [1.32–2.59], *p* = 0.0004, respectively) than later-year students. All students reported the main reasons for vaccination to be their own protection and parental decision. Concluded, present findings demonstrate a low prevalence of flu vaccination among medical students. Therefore, strategies are needed to increase the uptake of influenza vaccine in students, especially considering the future contact between this group of future health care workers and higher risk groups.

## 1. Introduction 

Influenza is a highly contagious viral respiratory tract infection prevented with influenza vaccines. Due to the frequent influenza virus mutations, the World Health Organization (WHO) gives new recommendations on the composition of the seasonal of influenza virus vaccines twice a year, for the northern hemisphere in February and southern one in September [1]. However, despite the regular introduction of new vaccines and growing vaccination uptake, the virus has not yet been eradicated: it remains in the top 10 most common global diseases in the 21st century. The WHO estimates one billion cases of influenza annually, 3–5 million of which are severe, and from 290,000 to 650,000 fatal ones, including 72,000 in the WHO Europe Region [2,3].

The National Influenza Center, in the National Institute of Public Health-National Institute of Hygiene (NIZP-PZH), is responsible for epidemic surveillance in Poland and publishes epidemiological weekly reports on influenza and influenza-like illness, hospitalizations, and deaths from influenza [4]. According to these reports, the incidence of influenza in Poland has remained high for a while.

Unfortunately, influenza is often confused with the common cold by patients themselves or even medical students/staff, which influence the treatment. The two diseases differ in causes, symptoms, and above all serious consequences such as pneumonia, exacerbations of chronic diseases requiring hospitalization, neurological complications, and even death [2,5]. It can be difficult to distinguish the symptoms of flu from colds, and some infections can be asymptomatic [6], but an infected subject can nevertheless act as a source of infection to others [7]. Thankfully, serious flu complications can be avoided by regular annual vaccination [2,5].

Flu vaccination programs play an important role in maintaining the high rate of coverage in the country, especially among people of higher risk groups; this reduces the risk of viral spread and the number of flu serious consequences, including hospitalization and death [8]. In addition, unchecked national epidemics may quickly become a global pandemic with devastating effects on health and social functioning. The WHO publishes annual updates on influenza vaccination. Nowadays, pregnant women are given the highest priority, followed by children aged 6–59 months, seniors, people with chronic diseases, and healthcare professionals [9,10]. As healthcare professionals, including students during clinical rotations, most often have contact with these risk groups, they should be responsible and aware of the need for annual immunization against influenza.

The WHO and Centers for Disease and Control Prevention (CDC) regard medical professionals as not only doctors, but also health-care workers (HCWs), such as medical students, nurses, midwives, medical assistants, pharmacists, public health assistants, and care workers in nursing homes for people with chronic medical conditions. Their work with people in high-risk and higher risk groups require them to receive annual vaccination to work safely (protect themselves, their relatives, co-workers, and patients) [9]. However, despite these recommendations, and those of The Pan American Health Organization (PAHO), European Center for Disease and Control Prevention (ECDC), Polish Ministry of Health and Social Welfare and Polish scientific societies and experts [5,9], the coverage of seasonal influenza vaccination rate for the entire population in Poland is one of the lowest among the European Union countries, being 3.4% in the 2015–2016 season and 3.3% in the 2016–2017 season [11]. A study of 11 European countries showed that in 2007/2008, vaccination coverage levels in the general population were 9.5% in Poland and 28.7% in the UK [12]. Similar data from the Organization for Economic Cooperation and Development (OECD) indicated low rate of vaccination in the group of seniors aged over 65 years in Poland: 6.89% in 2017, 6.97% in 2016, 9.7% in 2014, and 12.10% in 2009 [11,13]. These values are nevertheless higher than those in season 2012/2013, when coverage among outpatients aged 60–67 years with chronic diseases was only 4.4%, and in 2013/2014 3.16% [14]. Moreover, the influenza immunization rate in Poland is not only lower than WHO recommendations, i.e., 75%, but also lower than the mean European Union value (44%) [15]. Although only South Korea has managed to achieve the vaccination coverage rate recommended by the WHO, with a coverage of 83% [3], other countries are close to achieving this result: United Kingdom (72%), Chile (68.3%), United States (68.2%), New Zealand (62%) [13]. These results are more satisfactory than in Poland.

High flu vaccination coverage among health care workers (HCWs) promotes a high vaccination rate in the general population [16,17]. The range of influenza vaccination among HCWs has been found to be 82% of doctors and 42% of nurses in USA [18], 45% of doctors and 15% of nurses in Italy [19], 31% of doctors and 22% nurses in Germany [20], 48% of doctors and 31% of nurses in France [21], 66% of medical staff in Singapore [22], and 36% of medical staff in the Netherlands [23].

Coverage among Polish HCWs is slightly higher than in the general population, estimated to range from 5 to 10% in nurses [24,25], and approximately 22.3% in doctors [25]. Similarly, among medical students, coverage is ranging from 3 to 24%, depending on the major and year of study [26,27]. Few studies have examined coverage among Polish students and health care workers; these mainly deal with the problem of flu vaccination among medicine students, and related to one year of study and one or two majors of study. This study is the first to address influenza vaccination coverage among students of four majors of study at all years of their study, at a Medical University in Poland.

The aim of the study is to compare the rates of influenza immunization in students in four majors of study: Nursing, Midwifery, Pharmacy, and Public health in all years of study; it also examines how their attitudes to influenza prevention are changing with the year of study, and the reasons for receiving influenza vaccination, and determines the knowledge held by students for distinguishing flu from common cold symptoms.

## 2. Material and Methods

### 2.1. Participants

A cross-sectional survey was conducted in the period running 1st of December 2019 to the end of February 2020 in the Medical University in Lodz, the largest medical college in central Poland. An anonymous, self-administered survey was collected among students from four majors: Nursing, Midwifery, Pharmacy, and Public health students. The study was conducted on all years of study. The entire study period covers the years from the first to third years for majors of study: Nursing, Midwifery, and Public health, and the entire study period for major of Pharmacy includes the years from the first to the fifth year. A total of 1313 students were administered the survey.

Firstly, before completing the survey, participants were informed about the purpose of the study and how it is important for the prevention of influenza. Secondly, they were asked for permission to participate in the study. Then students were asked to complete the paper version survey accurately. Receiving back a blank survey was interpreted as not agreeing to participate in the study. A total of 1188 completed surveys were obtained, i.e., a 90.5% response rate. The study received approval at the Research Ethics Board in Medical University in Lodz, Poland number No RNN/141/13/KB.

### 2.2. Study Questionnaire

The study used an original questionnaire authored by the main researcher (Sylwia Kałucka, Head of the project) and a team of medical students from the Scientific Circle of the Medical University of Lodz. The study design was approved by the Ethics Committee of the Medical University of Lodz (No. RNN/141/13/KB).

The questionnaire was first administered to medical students and primary care patients in the flu seasons 2012–2013 and 2013–2014. The results were presented at the National Conference on Family Medicine, where they were awarded first place at the National Conference in Poland in 2014. The results of both studies have also been published [14,27]. The questionnaire has been repeatedly made available for Bachelor’s or Master’s theses in medical majors in Poland. An improved version of the questionnaire was used in the study. The questionnaire has been repeatedly used to assess the approach of respondents to the prevention of influenza, knowledge of the flu vaccine and viral respiratory infections.

This questionnaire includes 31 items in four main parts: 19 single answer questions, eight multiple-choice questions and four open-ended questions. The first part of the questionnaire enquires about the attitudes towards the seasonal influenza vaccine and vaccination rate. This part also includes one multi-choice question about how to differentiate a cold from flu, with seven answers: (1) a cold starts slowly, a flu appears suddenly (correct), (2) a flu starts slowly, a cold appears suddenly (incorrect), (3) fever is higher during flu than in a cold (correct), (4) muscle pain always occurs during a cold, never during flu (incorrect), (5) rhinitis is more common in the cold (correct), (6) nosebleeds occur frequently in the flu (incorrect) or (7) it is hard to say (incorrect). The following scoring system was used: zero to one correct answer indicates very poor/poor knowledge; two to three correct answers indicates rather good/very good knowledge; zero to two incorrect answers indicates not bad knowledge, three to four incorrect answers indicates very bad knowledge. Part one also includes an open-ended question where a student was asked about the reason/reasons for the influenza vaccination and to give examples.

The second part includes a knowledge quiz on the influenza vaccine (e.g., how often one should be vaccinated) and its effectiveness and side effects. Other questions are multiple-choice and open-ended: for whom would the respondent particularly recommend flu vaccination (e.g., high-risk groups). The third part consists of information about the health of the respondent, and the fourth part collects demographic information: sex, age, major of study and year of study (among students), place of residence, and also cigarette smoking status (current, ex-smoker, never smoking).

### 2.3. Statistical Data

The data were analyzed by descriptive statistics using STATISTICA 13.1 (Stat Soft Inc., Krakow, Poland). Means and standard deviations were calculated for measurable variables, and percentage frequency of occurrence was given for qualitative features. The relationships between factors influencing influenza vaccination and the level of influenza knowledge were compared using univariate and multivariate logistic regression analysis. Findings characterized by *p* < 0.05 in multivariate analysis were regarded as statistically significant; these variables were subjected to univariate analysis, in which significance was regarded as *p* < 0.1.

## 3. Results

### 3.1. Demographics

The survey was completed by 1188 participants, but fully completed questionnaires were obtained in 1137 students: 446 Nursing students (39.2%), 167 Midwifery students (14.7%), 436 Pharmacy students (38.3%), and 88 Public health students (7.7%). Incomplete questionnaires were discarded. Most students were female (100% of Midwifery, 94.0% of Nursing, 90.9% of Public health, and 83.7% of Pharmacy). Such a high percentage of women is typical of these disciplines in Poland. The mean age was 21.3 ± 1.62 SD years; no statistically significant age difference was observed between the majors (Table 1). Most Nursing, Midwifery, and Public health students were from rural backgrounds (35.7%, 33.6%, 33.0%, respectively), while most Pharmacy students were from large cities above 100,000 residents (55.9%) (Table 1).

Most students did not suffer from chronic diseases and did not smoke tobacco. The highest numbers of current smokers were found among Nursing students (12.6%), and of former smokers among Public health students (28.4%). The most frequently reported diseases according to major were asthma in Pharmacy students (4.4%), thyroid disease in Nursing and Midwifery students (4.0%, 6.0%, respectively), and immunity disorders in Public health students (6.8%). Taking medication for chronic disease was reported by 18.2% of Public health students, 16.7% of Nursing and Pharmacy students, and 16.8% of Midwifery students. (Table 1.)

### 3.2. Attitude of Influenza Vaccination

Our results indicate that Public health students were the most often vaccinated against influenza (48.9%) (Table S1), followed by Pharmacy and Nursing students (31%, 30.7%, respectively) (Tables S2 and S3) and least often Midwifery students (25.1%) (Table S4). In all majors, male students were vaccinated much more often than female students (Tables S1–S4): each year, 3.7% of male Nursing students and 1.2% of female Nursing students became vaccinated, 8.5% of male Pharmacy vs. 1.4% female students and 12.5% male Public health students vs. 0.0% female students. Male students also significantly more often occasional (not regularly every year) vaccinated: men 22.2% vs. women 8.4% in Nursing; men 25.4% vs. women 14.0% in Pharmacy; men 37.5% vs. women 36.3% in Public health (Tables S1–S3). In all four majors, the numbers of vaccinations fell as the year of study increased (Tables S1–S4).

Regarding place of residence, 76.7% of Nursing and 77.1% of Pharmacy students living in rural areas were not vaccinated (Tables S2 and S3). Among the students in the urban areas, 77.3% of Midwifery students in cities with 10,000–100,000 inhabitants and 56.5% of Public health students in cities above 100,000 residents were not vaccinated (Tables S1–S4). Non-smokers were more likely to be unvaccinated than smokers among Midwifery students (non-smokers—73.8% vs. smokers—66.7%), Pharmacy students (non-smokers—72.0% vs. smokers—47.5%), and Public health students (non-smokers—50.9% vs. smokers—37.5%) (Tables S1, S2 and S4). In contrast, among Nursing students, non-smokers were vaccinated more often than smokers (unvaccinated, respectively, 71.7% and 76%), but this is not a statistically significant difference (Table S3).

In Midwifery, Pharmacy, and Public health, students suffering from chronic diseases were vaccinated less frequently against influenza (percent unvaccinated: 85.7%, 71.0%, 57.9%, respectively) than total healthy students (72%, 68.7%, 49.3%, respectively). In Nursing, healthy students were more likely to be unvaccinated than chronically ill students (70.1% vs. 65.9%) (Tables S1–S4). In all majors, students who take medication chronically, are more likely to have never received a flu vaccination than those who do not. However, the difference is small and not statistically significant (Tables S1–S4).

The univariate and multivariate logistic regression analysis of the factors associated with taking seasonal influenza vaccinations are presented in Table 2. Four factors were found to be significantly associated with receiving seasonal influenza vaccinations among students. The first is male sex, where male students are vaccinated almost three times more often than female students (OR: 2.75; 95% CI [1.78–4.24], *p* = 0.000005). The second factor is studying Public health, whose students were almost 2.5 times more likely to be vaccinated against seasonal influenza than those of Nursing (OR: 2.37; 95% CI [1.46–3.85], *p* = 0.000498). The year of study also had a significant influence on influenza vaccination, with Year 1 students being almost three times more likely to be vaccinated than senior students, i.e., in Years 3, 4, or 5 (OR: 2.75; 95% CI [1.99–3.82], *p* = 0.00000), and with the 2 Year students being nearly twice as likely (OR: 1.84; 95% CI [1.32–2.59], *p* = 0.0004). Finally, place of residence also had an influence: students living in medium-sized cities (i.e., 10,000–100,000 residents) and larger cities (i.e., above 100,000 residents) were 1.5 times more likely to be vaccinated than those from rural areas (OR: 1.52; 95% CI [1.03–2.24], *p* = 0.035085, OR: 1.52; 95% CI [1.10–2.10], *p* = 0.010362, respectively). In both cases, living in a larger city resulted that students vaccinated against flu 1.5 times more often (Table 2).

### 3.3. The Reasons for Influenza Vaccination among the Subjects

The two most common reasons given by all students for being vaccinated were personal protection and parental decision. Personal protection was most commonly indicated by Pharmacy students and Public health students (64.4%, 62.8%, respectively), followed by Midwifery students (50.0%) and then Nursing students (37.6%). Parental decision was most often reported by Nursing students (22.6%), followed by Midwifery, Pharmacy, and Public health students (16.6%, 15.0%, 14.0%, respectively) (Table 3).

### 3.4. Knowledge about Symptom Difference Common Cold and Influenza among the Subjects

The highest numbers of incorrect answers were given by Pharmacy students, both those vaccinated against influenza (1.5%) and unvaccinated (2.7%) (Table S5). However, vaccinated students generally chose fewer correct answers than unvaccinated students (Midwifery—42.8% vs. 66.4%; Pharmacy—40.8% vs. 58.5%; Nursing—32.1% vs. 46.7%; Public health—30.2% vs. 62.3%). The unvaccinated students demonstrated much better knowledge in distinguishing flu symptoms from colds than the vaccinated students. The statistical data are included in Table S5.

The univariate and multivariate logistic regressions analyses regarding knowledge of flu and cold symptoms are given in Table 4. Four factors were found to be statistically significant: year of study, non-vaccination against influenza, residence, and smoking. Higher year students (years 3–5) gave better answers regarding the basic symptoms of flu and colds than first year students (OR: 1.35; 95% CI [1.00–1.83], *p* = 0.05), unvaccinated students were significantly more likely to give correct answers than vaccinated ones (OR: 2.31; 95% CI [1.76–3.03], *p* = 0.000000), rural residents were more likely to answer correctly than those living in larger cities above 100,000 residents (OR: 1.72; 95% CI [1.28–2.31], *p* = 0.000309), and current smokers were more than twice as likely to give correct answers than non-smoking students (OR: 2.16; 95% CI [1.42–3.31], *p* = 0.000391) (Table 4).

## 4. Discussion

The present study examined the attitudes and behaviors towards seasonal influenza vaccination among students of a Medical University and evaluated their knowledge about influenza and the common cold. Unlike previous studies in this area, it surveyed a large group of students from four medical majors (Nursing, Midwifery, Pharmacy, and Public Health) and all years of study.

Few studies have examined influenza vaccination among medical school students in Poland, and these have usually included a small number of students [28,29,30] from one or two medical majors of study [26,27,28,29] and from one to three years of study [25,27,30,31,32,33]; in addition, they have typically been restricted to one or two epidemic seasons [25,27,29,31,32,33] and mainly concerned medicine students. They report a wide range of data regarding regular vaccination by Medical University students, ranging from 3 to 24.6% in Lublin [26,28], from 8.7 to 15.8% in Wroclaw [29,32], from 13.4 to 17.1% in Warsaw [25,33], and 24.3% in Lodz [27].

Our findings indicate that the level of vaccination differs between majors of study. Public health students were most likely to vaccinate against flu (48.9%), followed by Pharmacy and Nursing (31%, 30.7%, respectively), and then Midwifery (25.1%). However, too few students were vaccinated regularly annually. Male students were significantly more likely to declare being vaccinated than female students [34]; however, the significant disparity between the numbers of male and female participants must be taken into consideration, with women predominating in all these professions (especially in Midwifery and Nursing). 

The prevalence of influenza vaccination among the studied population is far below global recommendations. WHO guidelines recommend that 75% of HCWs should be vaccinated [9]. Although few countries manage to achieve this rate of coverage among medical students, much higher levels were noted in Canada with 71.2% being regularly vaccinated, including students of Medicine (86.3%), Nursing (52.4%), and Pharmacy (67.7%). In Australia, 53.8% of medical students are vaccinated [17]. In contrast, similar levels to Poland have been noted among Hong Kong Nursing students (15.2%) [35], in Spain (5.3% of Nursing students) [36], Italy (less than one third of students) [37], and Slovakia, with only one quarter of students having been vaccinated [38]. In China, less than 10% have been vaccinated for three consecutive seasons [39].

In the present study, the most common reason for vaccination of students of all majors of study was self-protection, just like students in Canada, Saudi Arabia, Italy, Spain, and health care workers in Great Britain. The next most common was parental decision, followed by frequent infection of upper respiratory tract and by protection of others (family/patients) as shown also in other research [17,19,34,36,40]. Another important role in the decision about vaccination was played by *buzzy marketing* i.e., the opinions of immediate family members [41]; similar findings were found among the Polish population.

Vaccinated students mainly think about their own protection; this is a very positive finding because this indirectly protects their families, patients, and colleagues. It has been found that 25% of HCWs had contact with influenza viruses every year [7] and that 25% of medical staff have carried influenza virus infection confirmed by serological tests, but without any symptoms [6]. Therefore, it is important for HCWs to be allowed to receive the flu vaccine every year.

More disturbingly, since the third year of study, the desire to vaccinate against flu appeared to fall, so, it was the lowest in the years of study when students spent more time on clinical practice and working with patients than spending time on lectures. A similar pattern has been reported in studies from Spain, Canada, and China [16,39,42]. In contrast, later-year students have been found to be more frequently vaccinated than younger ones in Saudi Arabia and Australia [17,34].

This may be due to the fact that students rarely suffer from chronic diseases, feel healthy, and have no support in receiving free vaccinations. However, the combination of a lack of influenza vaccination and a mild course of viral infection can result in a high chance of infection for patients. The crowded study and living environment, and high mobility (e.g. campus, lectures, various activities), characterizing university life favors the fact transmission of respiratory viral diseases through the student community [39]. A growing body of research indicates that vaccination of medical students and healthcare professionals has a positive effect on reducing the transmission of infection [5,43,44]. It is well established that vaccination in young people is more effective (70–90% effective) than in older people (30–40% effective) [45,46]. It has been found that influenza vaccination for HCWs can reduce the risk of influenza virus infection by 88–89% [47,48]; there is also a strong case for vaccinating students against influenza before clinical practice, as after vaccination, the percentage of HCWs with influenza was found to fall from 42 to 9%, confirmed by laboratory tests, and hospital infections fell from 32 to 3% [49].

Sometimes distinguishing influenza infection from simple common cold is not easy [5]. However, our findings are optimistic, because students of later years of study appear to have greater knowledge in this regard than their younger colleagues, regardless of the major of study; this was also found for unvaccinated students compared to vaccinated ones, in contrast to other research [50,51]. This suggests that education at university may be at a high intellectual level, but this does not go hand in hand with the development of awareness of responsibility for others (empathetic attitude) or an ethical attitude, a lack of which can be observed among medical staff. For example, the presence of subtle or mild symptoms, associated with a mild course of influenza may not prevent the HCW from going to work [5,6]. Two cases have been described in a neonatal intensive care unit [52] and a bone marrow transplant unit [53]: in the former, 19 of the 54 newborns caught influenza and one died, while in the latter, 10 patients were affected, and one died. In these cases, only 12% and 15% of HCWs in these units were vaccinated against influenza, and continued to look after the patients when they had symptoms of a respiratory infection.

In the current study presented attitude for influenza vaccination among students of Nursing, Midwifery, Pharmacy, and Public health. As future HCWs, they will constitute a larger professional group than doctors alone, and with be in contact with a much wider cross-section of society, particularly the pharmacists and Public health practitioners. 

Medical studies prepare students to educate society on health matters. However, to popularize prevention by society, students must first begin with themselves, and one such step is vaccination against influenza. Educational programs regarding vaccination in medical schools are intended to increase knowledge, the ability to talk about potential side effects, positive attitudes to vaccination, and building immunization confidence [54,55], because vaccinated students and HCWs have higher knowledge of the vaccine and influenza than unvaccinated ones [50,51]. In the US, Public health students have taken part in programs to educate kindergarten parents and caregivers about the influenza virus and the influenza vaccine; this intervention resulted in greater compliance for vaccination among prekindergarten children and decreased absence [56]. Students of Public health, Nursing, Midwifery, and Pharmacy can also engage in education in educational care facilities [57,58], but also in hard-to-reach social groups [59]. The disadvantaged and the homeless have many problems with the use of prophylaxis [60,61]; however, understanding the specific health needs of homeless people has been found to have a generally positive influence on vaccination uptake, doubling the rate within this group [59]. Similarly, assisting with the education of other social groups during the course of Midwifery studies may be particularly useful in improving communication with pregnant women and their families: in the present study, this group had the lowest rate of vaccination. Midwives play a key role in implementing the influenza vaccination for pregnant women. WHO recommends seasonal influenza vaccination for pregnant women as a higher-risk group for flu complications [9]. A study in Taiwan found that low acceptance of the flu vaccination among pregnant women can change as medical staff increases their competence before they would perform the influenza vaccine during pregnancy [62] as is also in France [63]. 

A study in Canada examined the possibility of performing training for administering independent vaccinations during pre-Nursing practice. Following the intervention, the students perceived themselves as more confident and capable of providing safe vaccination [64]. Such training is arguably necessary when permitting vaccination without a medical prescription: in France, nurses have been able to vaccinate patients against influenza without medical prescription since 2008 to improve vaccination coverage. Three-quarters of Nursing students were positive about this [65].

Vaccination against seasonal flu is different from all other vaccinations offered. It must be repeated annually. Therefore, maintaining a high rate of flu vaccination coverage in population is a huge challenge for public health. Although vaccination against influenza is not mandatory in Poland, it is still recommended by the Polish Ministry of Health and many scientific societies [5]. Our findings indicate that the ratio of flu vaccination levels among medical students is higher than in the general population. In addition, vaccination rates are also higher among HCWs in Poland [66] than in the general population (8.2% in 2012/2013, 3.4% in 2015/2016), but still at a very low level (below 10%) [42,67].

## 5. Conclusions

Our findings demonstrate a low prevalence of flu vaccination among medical students in Midwifery, Nursing, Pharmacy, and Public health, and a poor understanding of the difference between influenza and the common cold. More effective education regarding influenza vaccination is needed to increase the coverage of vaccines in future healthcare workers. Greater efforts are needed to promote vaccine awareness among midwives, nurses, pharmacists, and public health assistants.

## Figures and Tables

**Table 1:** Study population demographics and general characteristics (N = 1137).

Group Characteristics	Major			
	Nursing N = 446 (39.2%)	Midwifery N = 167 (14.7%)	Pharmacy N = 436 (38.3%)	Public Health N = 88 (7.7%)
**Sex**				
Female	419 (94.0%)	167 (100.0%)	365 (83.7%)	80 (90.9%)
Male	27 (6.0%)	0 (0.0%)	71 (16.3%)	8 (9.1%)
**Age, mean ± SD**	21.1 ± 1.60	21.0 ± 1.43	21.6 ± 1.71	21.4 ± 1.49
18–20 y	167 (37.4%)	63 (37.7%)	136 (31.2%)	30 (34.1%)
21–22 y	220 (49.3%)	88 (52.7%)	178 (40.8%)	31 (35.2%)
23–24 y	38 (8.6%)	11 (6.6%)	106 (24.3%)	26 (29.6%)
≥25 y	21 (4.7%)	5 (3.0%)	16 (3.7%)	1 (1.1%)
**Year of Study**				
1st	182 (40.8%)	52 (40.8%)	118 (27.1%)	30 (34.1%)
2nd	150 (33.6%)	60 (33.6%)	106 (24.3%)	27 (30.7%)
3rd	114 (25.6%)	55 (25.6%)	87 (19.9%)	31 (35.2%)
4th	NA	NA	71 (16.3%)	NA
5th	NA	NA	54 (12.4%)	NA
**Place of residence**				
Rural	159 (35.7%)	56 (33.6%)	118 (27.1%)	29 (33.0%)
City of less than 10,000 r	27 (6.0%)	13 (7.8%)	51 (11.7%)	12 (13.6%)
City from 10,000 to 100,000 r	113 (25.3%)	44 (26.3%)	23 (5.3%)	24 (27.3%)
City above 100,000 r	147 (33.0%)	54 (32.3%)	244 (55.9%)	23 (26.1%)
**Cigarette smoking**				
Current smoker	56 (12.6%)	12 (7.2%)	40 (9.1%)	8 (9.1%)
Never smoker	318 (71.3%)	122 (73.0%)	353 (81.0%)	55 (62.5%)
Ex-smoker	72 (16.1%)	33 (19.8%)	43 (9.9%)	25 (28.4%)
**Status health—chronic disease**				
No, any	358 (80.3%)	132 (79.0%)	367(84.2%)	69 (78.4%)
**Yes (total)**	88 (19.7%)	35 (21.0%)	69 (15.8%)	19 (21.6%)
Asthma	13 (2.9%)	3 (1.8%)	19 (4.4%)	1 (1.1%)
Allergy	8 (1.8%)	4 (2.4%)	6 (1.4%)	1 (1.1%)
Immune disorders	11 (2.5%)	2 (1.2%)	7 (1.6%)	6 (6.8%)
Thyroid disease	18 (4.0%)	10 (6.0%)	9 (2.1%)	5 (5.7%)
Diabetes	2 (0.4%)	0 (0.0%)	6 (1.4%)	1 (1.1%)
Others	52 (11.7%)	26 (15.6%)	47 (10.8%)	14 (15.9%)
**Taking medication for chronic disease**				
No	359 (83.3%)	139 (83.2%)	363 (83.3%)	72 (81.8%)
Yes	87 (16.7%)	28 (16.8%)	73 (16.7%)	16 (18.2%)

r-residents, NA-not applicable.

**Table 2:** Associations between characteristics subjects (sex, age, major, year of study, place of residence, smoking cigarette, status health, taking medication) and vaccinated students.

	Total	Vaccinated	Univariate Logistic Regression		Multivariate Logistic Regression	
			OR 95%CI	*p*	OR 95%CI	*p*
**Sex**						
Female	1031 (90.7%)	300 (29.1%)	1.00 Ref.		1.00 Ref.	
Male	106 (9.3%)	57 (53.8%)	2.83 (1.89–4.25)	0.000001	2.75 (1.78–4.24)	0.000005
**Age**						
18–20 y	396 (34.8%)	152 (38.4%)	1.81 (0.89–3.71)	0.103161		
21–22 y	517 (45.5%)	142 (27.5%)	1.10 (0.54–2.25)	0.789982		
23–24 y	181 (15.9%)	52 (28.7%)	1.17 (0.55–2.50)	0.680115		
≥25 y	43 (3.8%)	11 (25.6%)	1.00 Ref.			
**Major**						
Nursing	446 (39.2%)	137 (30.7%)	1.00 Ref.		1.00 Ref.	
Midwifery	167 (14.7%)	42 (25.1%)	0.76 (0.51–1.13)	0.178146	0.87 (0.57–1.31)	0.493672
Pharmacy	436 (38.3%)	135 (31.0%)	1.01 (0.76–1.35)	0.937019	1.05 (0.77–1.45)	0.741444
Public health	88 (7.7%)	43 (48.9%)	2.16 (1.35–3.43)	0.001211	2.37 (1.46–3.85)	0.000498
**Year of study**						
1st	382 (33.6%)	157 (41.1%)	2.54 (1.86–3.47)	0.000000	2.75 (1.99–3.82)	0.000000
2nd	342 (30.1%)	111 (32.5%)	1.75 (1.26–2.42)	0.000798	1.84 (1.32–2.59)	0.000400
3rd + 4th + 5th	413 (36.2%)	89 (21.5%)	1.00 Ref.		1.00 Ref	
**Place of residence**						
Rural	362 (31.8%)	94 (26.0%)	1.00 Ref.		1.00 Ref.	
City of less than 10,000 r	103 (9.1%)	38 (36.9%)	1.67 (1.05–2.65)	0.031158	1.43 (0.87–2.33)	0.155302
City from 10,000 to 100,000 r	204 (17.9%)	70 (34.3%)	1.49 (1.03–2.16)	0.036289	1.52 (1.03–2.24)	0.035085
City above 100,000 r	468 (41.2%)	155 (33.1%)	1.41 (1.04–1.91)	0.026229	1.52 (1.10–2.10)	0.010362
**Cigarette smoking**						
Current smoker	848 (74.6%)	248 (29.2%)	1.00 Ref			
Never smoker	116 (10.2%)	43 (37.1%)	0.70 (0.47–1.05)	0.086604		
Ex-smoker	173 (15.2%)	66 (38.2%)	1.05 (0.64–1.70)	0.852553		
**Status health—chronic disease**						
No, any	926 (81.4%)	294 (31.7%)	1.09 (0.79–1.51)	0.593311		
Yes	211 (18.6%)	63 (29.9%)	1.00 Ref.			
**Taking medication for chronic disease**						
No	933 (82.1%)	299 (32.0%)	1.19 (0.85–1.66)	0.314047		
Yes	204 (17.9%)	58 (28.4%)	1.00 Ref.			

Univariate logistic regression and multivariate logistic regression were used. The multivariate model included variables at a significance level of *p* < 0.05 in univariate analysis. r-residents.

**Table 3:** The reasons for influenza vaccination among the subjects.

Self-Choice Answer	Major			
	Nursing	Midwifery	Pharmacy	Public Health
Personal protection	50 (37.6%)	21 (50.0%)	82 (64.6%)	27 (62.8%)
Frequent respiratory infections	16 (12.0%)	5 (11.9%)	4 (3.1%)	4 (9.3%)
Previous flu infection	6 (4.5%)	2 (4.8%)	6 (4.7%)	4 (9.3%)
Protection of others	12 (9.0%)	5 (11.9%)	8 (6.3%)	2 (4.6%)
Parental decision	30 (22.6%)	7 (16.6%)	19 (15.0%)	6 (14.0%)
Doctor’s recommendation	11 (8.3%)	1 (2.4%)	6 (4.7%)	0 (0.0%)
Other	8 (6.0%)	1 (2.4%)	2 (1.6%)	0 (0.0%)

**Table 4:** Associations between students’ knowledge and sex, age, major, year of study, vaccinated or unvaccinated students, place of residence, smoking cigarette, status health, taking medication by the subjects.

	Total	Correct Answers	Univariate Logistic Regression		Multivariate Logistic Regression	
			OR 95%CI	*p*	OR 95%CI	*p*
**Sex**						
Female	1031 (90.7%)	542 (52.6%)	1.56 (1.04–2.35)	0.031796	1.30 (0.83–2.02)	0.249468
Male	106 (9.3%)	44 (41.5%)	1.00 Ref.		1.00 Ref.	
**Age**						
18–20 y	396 (34.8%)	190 (48.0%)	1.00 Ref.			
21–22 y	517 (45.5%)	276 (53.4%)	1.24 (0.96–1.61)	0.105881		
23–24 y	181 (15.9%)	98 (54.1%)	1.28 (0.90–1.82)	0.170115		
≥25 y	43 (3.8%)	22 (51.2%)	1.14 (0.60–2.13)	0.691801		
**Major**						
Nursing	446 (39.2%)	213 (47.8%)	1.05 (0.66–1.66)	0.841278	0.91 (0.56–1.48)	0.704006
Midwifery	167 (14.7%)	101 (60.5%)	1.75 (1.04–2.96)	0.034809	1.50 (0.87–2.59)	0.146911
Pharmacy	436 (38.3%)	231 (53.0%)	1.29 (0.82–2.05)	0.274755	1.29 (0.79–2.11)	0.315209
Public health	88 (7.7%)	41 (46.6%)	1.00 Ref.		1.00 Ref.	
**Year of study**						
1st	382 (33.6%)	176 (46.1%)	1.00 Ref.		1.00 Ref.	
2nd	342 (30.1%)	179 (52.3%)	1.29 (0.96–1.72)	0.092735	1.28 (0.94–1.74)	0.115440
3rd+4th+5th	413 (36.2%)	231 (55.9%)	1.49 (1.12–1.97)	0.005633	1.35 (1.00–1.83)	0.051204
**Vaccinated**						
Yes	357 (31.4%)	130 (36.4%)	1.00 Ref.		1.00 Ref.	
No	780 (68.6%)	456 (58.5%)	2.46 (1.90–3.18)	0.000000	2.31 (1.76–3.03)	0.000000
**Place of residence**						
Rural	362 (31.8%)	215 (59.4%)	1.65 (1.25–2.18)	0.000428	1.72 (1.28–2.31)	0.000309
City of less than 10,000 r	103 (9.1%)	45 (43.7%)	0.87 (0.57–1.34)	0.541153	0.97 (0.62–1.52)	0.894609
City from 10,000 to 100,000 r	204 (17.9%)	106 (52.0%)	1.22 (0.88–1.70)	0.238108	1.30 (0.91–1.87)	0.149560
City above 100,000 r	468 (41.2%)	220 (47.0%)	1.00 Ref.		1.00 Ref.	
**Cigarette smoking**						
Current smoker	848 (74.6%)	422 (49.8%)	1.00 Ref		1.00 Ref	
Never smoker	116 (10.2%)	74 (63.8%)	1.78 (1.19–2.66)	0.005071	2.16 (1.42–3.31)	0.000391
Ex-smoker	173 (15.2%)	90 (52.0%)	1.09 (0.79–1.52)	0.588295	1.29 (0.91–1.82)	0.157487
**Status health—chronic disease**						
No, any	926 (81.4%)	474 (51.2%)	1.00 Ref.			
Yes	211 (18.6%)	112 (53.1%)	1.08 (0.80–1.46)	0.619720		
**Taking medication for chronic disease**						
No	933 (82.1%)	471 (50.5%)	1.00 Ref.			
Yes	204 (17.9%)	115 (56.4%)	1.27 (0.93–1.72)	0.128076		

Univariate logistic regression and multivariate logistic regression were used. The multivariate model included variables at a significance level of *p* < 0.05 in univariate analysis. r-residents.

## Supplementary Materials

The following are available online at https://www.mdpi.com/2076-393X/8/3/516/s1, Table S1: Prevalence of vaccination stratified by sex, age, year of study, place of residence, smoking cigarette, status health, taking medication in Public health., Table S2: Prevalence of vaccination stratified by sex, age, year of study, place of residence, smoking cigarette, status health, taking medication in Pharmacy. Table S3: Prevalence of vaccination stratified by sex, age, year of study, place of residence, smoking cigarette, status health, taking medication in Nursing. Table S4: Prevalence of vaccination stratified by sex, age, year of study, place of residence, smoking cigarette, status health, taking medication in Midwifery. Table S5: Knowledge about influenza and common cold among unvaccinated and vaccinated students.
