# Supplementary material for: A Comparison of the Attitudes to Influenza Vaccination Held by Nursing, Midwifery, Pharmacy, and Public Health Students and Their Knowledge of Viral Infections
Source: Vaccines (Basel). 2020 Sep 9;8(3):516. doi: 10.3390/vaccines8030516 (PMC7565400; doi:10.3390/vaccines8030516)
Supplement: Supplementary file 1 [file vaccines-08-00516-s001.pdf]

Table S1. Prevalence of vaccination stratified by sex, age, year of study, place of residence, smoking cigarette, status health, taking medication in Public health

Influenza vaccination	No	Yes		
**Total**	**45 (51.1%)**	**43 (48.9%)**		
		Once	Irregularly*	Regularly**
**Sex**				
Female	44 (55.0%)	7 (8.8%)	29 (36.3%)	0 (0.0%)
Male	1 (12.5%)	3 (37.5%)	3 (37.5%)	1 (12.5%)
**Age**				
18-20y	11 (36.7%)	4 (13.3%)	15 (50.0%)	0 (0.0%)
21-22y	17 (54.8%)	5 (15.1%)	8 (25.8%)	1 (3.2%)
23-24y	17 (65.4%)	1 (3.8%)	8 (30.8%)	0 (0.0%)
≥25y	0 (0.0%)	0 (0.0%)	1 (100.0%)	0 (0.0%)
**Year of study**				
1st	8 (26.7%)	5 (16.7%)	17 (56.7%)	0 (0.0%)
2nd	17 (63.0%)	3 (11.1%)	6 (22.2%)	1 (3.7%)
3rd	20 (64.5%)	2 (6.5%)	9 (29.0%)	0 (0.0%)
4th	NA	NA	NA	NA
5th	NA	NA	NA	NA
**Place of residence**				
rural	13 (44.8%)	2 (6.9%)	13 (44.8%)	1 (3.4%)
city of less than 10,000 r	6 (50.0%)	0 (0.0%)	6 (50.0%)	0 (0.0%)
city from 10,000 to 100,000 r	13 (54.2%)	0 (0.0%)	11 (45.8%)	0 (0.0%)
city above 100,000 r	13 (56.5%)	8 (34.8%)	2 (8.7%)	0 (0.0%)
**Cigarette smoking**				
current smoker	3 (37.5%)	1 (12.5%)	4 (50.0%)	0 (0.0%)
never smoker	28 (50.9%)	6 (10.9%)	20 (36.4%)	1 (1.8%)
ex-smoker	14 (56.0%)	3 (12.0%)	8 (32.0%)	0 (0.0%)
**Status health –chronic disease**				
No, any	34 (49.3%)	8 (11.6%)	26 (37.7%)	1 (1.4%)
Yes (total)	11 (57.9%)	2 (10.5%)	6 (31.6%)	0 (0.0%)
Asthma	0 (0.0%)	1 (100.0%)	0 (0.0%)	0 (0.0%)
Allergy	1 (100.0%)	0 (0.0%)	0 (0.0%)	0 (0.0%)
Immune disorders	4 (66.7%)	0 (0.0%)	2 (33.3%)	0 (0.0%)
Thyroid disease	3 (60.0%)	1 (20.0%)	1 (20.0%)	0 (0.0%)
Diabetes	1 (100.0%)	0 (0.0%)	0 (0.0%)	0 (0.0%)
Others	9 (64.3%)	1 (7.1%)	4 (28.6%)	0 (0.0%)
**Taking medication for chronic disease**				
No	36 (50.0%)	8 (11.1%)	27 (37.5%)	1 (1.4%)
Yes	9 (56.3%)	2 (12.5%)	5 (31.3%)	0 (0.0%)

* within last 3 years, ** minimum from 3 years, r - residents, NA - Not Applicable

Table S2. Prevalence of vaccination stratified by sex, age, year of study, place of residence, smoking cigarette, status health, taking medication in Pharmacy

**Influenza vaccination**	No	Yes		
**Total**	**301 (69%)**	**135 (31%)**		
		Once	Irregularly*	Regularly**
**Sex**				
Female	265 (72.6%)	44 (12.1%)	51 (14.0%)	5 (1.4%)
Male	36 (50.7%)	11 (15.5%)	18 (25.4%)	6(8.5%)
**Age**				
18-20y	84 (61.8%)	28 (20.6%)	22 (16.2%)	2 (1.5%)
21-22y	123 (69.1%)	19 (10.7%)	31 (17.4%)	5 (2.8%)
23-24y	84 (79.2%)	7 (6.6%)	13 (12.3%)	2 (1.9%)
≥25y	10 (62.5%)	1 (6.3%)	3 (18.8%)	2 (12.5%)
**Year of study**				
1st	71 (60.2%)	25 (21.2%)	20 (16.9%)	2 (1.7%)
2nd	68 (64.2%)	20 (18.9%)	14 (13.2%)	4 (3.8%)
3rd	63 (72.4%)	4 (4.6%)	18 (20.7%)	2 (2.3%)
4th	58 (81.7%)	3 (4.2%)	10 (14.1%)	0 (0.0%)
5th	41 (75.9%)	3 (5.6%)	7 (13.0%)	3 (5.6%)
**Place of residence**				
rural	91 (77.1%)	9 (7.6%)	18 (15.3%)	0 (0.0%)
city of less than 10,000 r	36 (70.6%)	8 (15.7%)	7 (13.7%)	0 (0.0%)
city from 10,000 to 100,000 r	13 (56.5%)	4 (17.4%)	4 (17.4%)	2 (8.7%)
city above 100,000 r	161 (66.0%)	34 (13.9%)	40 (16.4%)	9 (3.7%)
**Cigarette smoking**				
current smoker	19 (47.5%)	6 (15.0%)	15 (37.5%)	0 (0.0%)
never smoker	254 (72.0%)	43 (12.2%)	49 (13.9%)	7 (2.0%)
ex-smoker	28 (65.1%)	6 (14.0%)	5 (11.6%)	4 (9.3%)
**Status health –chronic disease**				
No, any	252 (68.7%)	51 (13.9%)	55 (15.0%)	9 (2.5%)
Yes (total)	49 (71.0%)	4 (5.8%)	14 (20.3%)	2 (2.9%)
Asthma	12 (63.2%)	2 (10.5%)	5 (26.3%)	0 (0.0%)
Allergy	5 (83.3%)	0 (0.0%)	1 (16.7%)	0 (0.0%)
Immune disorders	3 (42.9%)	2 (28.6%)	2 (28.6%)	0 (0.0%)
Thyroid disease	2 (22.2%)	0 (0.0%)	0 (0.0%)	7 (77.8%)
Diabetes	5 (83.3%)	0 (0.0%)	1 (16.7%)	0 (0.0%)
Others	38 (80.9%)	0 (0.0%)	7 (14.9%)	2 (4.3%)
**Taking medication for chronic disease**				
No	249 (68.6%)	48 (13.2%)	57 (15.7%)	9 (2.5%)
Yes	52 (71.2%)	7 (9.6%)	12 (16.4%)	2 (2.7%)

* within last 3 years, ** minimum from 3 years, r - residents, NA - Not Applicable

Table S3. Prevalence of vaccination stratified by sex, age, year of study, place of residence, smoking cigarette, status health, taking medication in Nursing

**Influenza vaccination**	No	Yes		
**Total**	**309 (69.3%)**	**137 (30.7%)**		
		Once	Irregularly*	Regularly**
**Sex**				
Female	297 (70.9%)	82 (19.6%)	35 (8.4%)	5 (1.2%)
Male	12 (44.4%)	8 (29.6%)	6 (22.2%)	1 (3.7%)
**Age**				
18-20y	108 (64.7%)	42 (25.1%)	16 (9.6%)	1 (0.6%)
21-22y	163 (74.1%)	42 (19.1%)	13 (5.9%)	2 (0.9%)
23-24y	20 (52.6%)	5 (13.2%)	11 (28.9%)	2 (5.3%)
≥25y	18 (85.7%)	1 (4.8%)	1 (4.8%)	1 (4.8%)
**Year of study**				
1st	113 (62.1%)	46 (25.3%)	21 (11.5%)	2 (1.1%)
2nd	100 (66.7%)	36 (24.0%)	10 (6.7%)	4 (2.7%)
3rd	96 (84.2%)	8 (7.0%)	10 (8.8%)	0 (0.0%)
4th	NA	NA	NA	NA
5th	NA	NA	NA	NA
**Place of residence**				
rural	122 (76.7%)	21 (13.2%)	14 (8.8%)	2 (1.3%)
city of less than 10,000 r	14 (51.9%)	13 (48.1%)	0 (0.0%)	0 (0.0%)
city from 10,000 to 100,000 r	74 (65.5%)	30 (26.5%)	9 (8.0%)	0 (0.0%)
city above 100,000 r	99 (67.3%)	26 (17.7%)	18 (12.2%)	4 (2.7%)
**Cigarette smoking**				
current smoker	43 (76.8%)	6 (10.7%)	4 (7.1%)	3 (5.4%)
never smoker	228 (71.7%)	67 (21.1%)	21 (6.6%)	2 (0.6%)
ex-smoker	38 (52.8%)	17 (23.6%)	16 (22.2%)	1 (1.4%)
**Status health –chronic disease**				
No, any	251 (70.1%)	74 (20.7%)	32 (8.9%)	1 (0.3%)
Yes (total)	58 (65.9%)	16 (18.2%)	9 (10.2%)	5 (5.7%)
Asthma	7 (53.8%)	0 (0.0%)	4 (30.8%)	2 (15.4%)
Allergy	6 (75.0%)	2 (25.0%)	0 (0.0%)	0 (0.0%)
Immune disorders	4 (36.4%)	4 (36.4%)	2 (18.2%)	1 (9.1%)
Thyroid disease	12 (66.7%)	3 (16.7%)	1 (5.6%)	2 (11.1%)
Diabetes	1 (50.0%)	0 (0.0%)	1 (50.0%)	0 (0.0%)
Others	40 (68.3%)	8 (15.4%)	2 (3.8%)	2 (3.8%)
**Taking medication for chronic disease**				
No	248 (69.1%)	76 (21.2%)	33 (9.2%)	2 (0.6%)
Yes	61 (70.1%)	14 (16.1%)	8 (9.2%)	4 (4.6%)

* within last 3 years, ** minimum from 3 years, r - residents, NA - Not Applicable

Table S4. Prevalence of vaccination stratified by sex, age, year of study, place of residence, smoking cigarette, status health, taking medication in Midwifery

**Influenza vaccination**	No	Yes		
**Total**	**125 (74.9%)**	**42 (25.1%)**		
		Once	Irregularly*	Regularly**
**Sex**				
Female	125 (74.9%)	20 (12.0%)	18 (10.8%)	4 (2.4%)
Male	0 (0.0%)	0 (0.0%)	0 (0.0%)	0 (0.0%)
**Age**				
18-20y	41 (65.1%)	11 (17.5%)	10 (15.9%)	1 (1.6%)
21-22y	72 (81.8%)	9 (10.2%)	5 (5.7%)	2 (2.3%)
23-24y	8 (72.7%)	0 (0.0%)	2 (18.2%)	1 (9.1%)
≥25y	4 (80.0%)	0 (0.0%)	1 (20.0%)	0 (0.0%)
**Year of study**				
1st	33 (63.5%)	8 (15.4%)	9 (17.3%)	2 (3.8%)
2nd	46 (76.7%)	12 (20.0%)	2 (3.3%)	0 (0.0%)
3rd	46 (83.6%)	0 (0.0%)	7 (12.7%)	2 (3.6%)
4th	NA	NA	NA	NA
5th	NA	NA	NA	NA
**Place of residence**				
rural	42 (75.0%)	5 (8.9%)	7 (12.5%)	2 (3.6%)
city of less than 10,000 r	9 (69.2%)	3 (23.1%)	1 (7.7%)	0 (0.0%)
city from 10,000 to 100,000 r	34 (77.3%)	5 (11.4%)	4 (9.1%)	1 (2.3%)
city above 100,000 r	40 (74.1%)	7 (13.0%)	6 (11.1%)	1 (1.9%)
**Cigarette smoking**				
current smoker	8 (66.7%)	1 (8.3%)	1 (8.3%)	2 (16.7%)
never smoker	90 (73.8%)	15 (12.3%)	15 (12.3%)	2 (1.6%)
ex-smoker	27 (81.8%)	4 (12.1%)	2 (6.1%)	0 (0.0%)
**Status health –chronic disease**				
No, any	95 (72.0%)	17 (12.9%)	16 (12.1%)	4 (3.0%)
Yes (total)	30 (85.7%)	3 (8.6%)	2 (5.7%)	0 (0.0%)
Asthma	3 (100.0%)	0 (0.0%)	0 (0.0%)	0 (0.0%)
Allergy	3 (75.0%)	1 (25.0%)	0 (0.0%)	0 (0.0%)
Immune disorders	1 (50.0%)	0 (0.0%)	1 (50.0%)	0 (0.0%)
Thyroid disease	8 (80.0%)	1 (10.0%)	1 (10.0%)	0 (0.0%)
Diabetes	0 (0.0%)	0 (0.0%)	0 (0.0%)	0 (0.0%)
Others	22 (84.6%)	2 (7.7%)	2 (7.7%)	0 (0.0%)
**Taking medication for chronic disease**				
No	101 (72.7%)	18 (2.9%)	16 (11.5%)	4 (2.9%)
Yes	24 (85.7%)	2 (7.1%)	2 (7.1%)	0 (0.0%)

* within last 3 years, ** minimum from 3 years, r - residents, NA - Not Applicable

Table S5. Knowledge about influenza and common cold among unvaccinated and vaccinated students.

Self-choose answer	Major							
	Nursing		Midwifery		Pharmacy		Public health	
	vaccinated	unvaccinated	vaccinated	unvaccinated	vaccinated	unvaccinated	vaccinated	unvaccinated
**Number of correct answers**								
0-1 correct answers	93 (67.9%)	140 (53.3%)	24 (57.2%)	42 (33.6%)	80 (59.2%)	125 (41.5%)	28 (69.8%)	17 (37.7%)
2-3 correct answers	44 (32.1%)	169 (46.7%)	18 (42.8%)	83 (66.4%)	55 (40.8%)	176 (58.5%)	13 (30.2%)	28 (62.3%)
**Number of wrong answers**								
0-1-2 wrong answers	137 (100%)	308 (99,67%)	42 (100%)	125 (100%)	133 (98.5%)	293 (97.3%)	43(100%)	45 (100%)
3-4 wrong answers	0 (0.0%)	1 (0.3%)	0 (0.0%)	0 (0.0%)	2 (1.5%)	8 (2.7%)	0 (0.0%)	0 (0.0%)
